# Reasons for participation and non-participation in a diabetes prevention trial among women with prior gestational diabetes mellitus (GDM)

**DOI:** 10.1186/1471-2288-14-13

**Published:** 2014-01-24

**Authors:** Jennifer J Infanti, Angela O’Dea, Irene Gibson, Brian E McGuire, John Newell, Liam G Glynn, Ciaran O’Neill, Susan B Connolly, Fidelma P Dunne

**Affiliations:** 1 School of Medicine, Clinical Science Institute, National University of Ireland Galway, Galway, Ireland; 2 Croí–The West of Ireland Cardiac Foundation, Croí Heart and Stroke Centre, Moyola Lane, Newcastle, Galway, Ireland; 3 School of Psychology, National University of Ireland Galway, University Road, Galway, Ireland; 4 HRB Clinical Research Facility, National University of Ireland Galway, University Road, Galway, Ireland; 5 Discipline of General Practice, National University of Ireland Galway, 1 Distillery Road, Galway, Ireland; 6 J.E. Cairnes School of Business & Economics, Cairnes Building, National University of Ireland Galway, Galway, Ireland; 7 Division of Cardiology Cardiothoracic and Thoracic Surgery, Imperial College Healthcare NHS Trust, London, UK

**Keywords:** Gestational diabetes mellitus, Prevention of type 2 diabetes, Barriers to participation in lifestyle intervention, Risk factor modification, Randomised controlled trial

## Abstract

**Background:**

Gestational diabetes mellitus (GDM) is a risk factor for the development of type 2 diabetes. Lifestyle intervention can prevent progression to type 2 diabetes in high risk populations. We designed a randomised controlled trial (RCT) to evaluate the effectiveness of an established lifestyle intervention compared to standard care for delaying diabetes onset in European women with recent GDM. Recruitment into the RCT was more challenging than anticipated with only 89 of 410 (22%) women agreeing to participate. This paper identifies factors that could enhance participation of the target population in future interventions.

**Methods:**

We hypothesised that women who agreed to participate would have higher diabetes risk profiles than those who declined, and secondly that it would be possible to predict participation on the bases of those risk factors. To test our hypothesis, we identified the subset of women for whom we had comprehensive data on diabetes risks factors 3-5 years following GDM, reducing the sample to 43 participants and 73 decliners. We considered established diabetes risk factors: smoking, daily fruit and vegetable intake, participation in exercise, family history of diabetes, glucose values and BMI scores on post-partum re-screens, use of insulin during pregnancy, and age at delivery. We also analysed narrative data from 156 decliners to further understand barriers to and facilitators of participation.

**Results:**

Two factors differentiated participants and decliners: age at delivery (with women older than 34 years being more likely to participate) and insulin use during pregnancy (with women requiring the use of insulin in pregnancy less likely to participate). Binary logistic regression confirmed that insulin use negatively affected the odds of participation. The most significant barriers to participation included the accessibility, affordability and practicality of the intervention.

**Conclusions:**

Women with recent GDM face multiple barriers to lifestyle change. Intervention designers should consider: (i) the practicalities of participation for this population, (ii) research designs that capitalise on motivational differences between participants, (iii) alleviating concerns about long-term diabetes management. We hope this work will support future researchers in developing interventions that are more relevant, effective and successful in recruiting the desired population.

**Trial registration:**

Current Controlled Trials ISRCTN41202110

## Background

The prevalence of gestational diabetes mellitus (GDM) is growing around the world [1,2], and the adverse maternal and neonatal implications of GDM are now well established [1,3-7]. GDM is associated with increased stress and depression during pregnancy [7] and can have long-term consequences for the mother and child, including increased risk of hospital admission, obesity, heart disease and type 2 diabetes in later life [8-14]. Indeed, Bellamy *et al.*[10] found that progression to type 2 diabetes is increased seven-fold in women with prior GDM compared to women with normal glucose tolerance (NGT) in pregnancy.

Since the 1990s, evidence has increasingly demonstrated that healthy lifestyle intervention and behaviour modification has the potential to delay if not prevent progression to type 2 diabetes in high risk populations [15-21]. For example, the American National Institutes of Health designed an intensive individualised intervention targeted at improving eating and physical activity habits, called the Diabetes Prevention Program (DPP). The DPP led to a 50% reduction in diabetes incidence in adults with pre-diabetes [18,21]. Similarly the Finnish Diabetes Prevention Study (FDPS) demonstrated that weight loss from lifestyle change reduces the incidence of type 2 diabetes in patients with pre-diabetes [19]. Other studies have shown lifestyle intervention to be at least as effective as pharmacological intervention in reducing the risk of type 2 diabetes in people with impaired glucose tolerance [22]. Lindstrom *et al.*[23] found that lifestyle changes are sustained and the incidence of diabetes reduced after the intervention stops.

There is limited evidence, however, to support the efficacy of diabetes prevention programmes in women with a history of GDM. The most compelling evidence in this subgroup comes from the DPP which showed a 50% reduction in diabetes incidence in women within 10 years of GDM [24]. Efforts to engage women with a more recent history of GDM in type 2 diabetes prevention efforts have largely proven challenging though, due to problems with uptake of screening services post-pregnancy [25,26] and recruitment and retention challenges in the case of behaviour modification interventions [27,28].

In fact, the literature suggests that women with prior GDM who are at highest risk of developing type 2 diabetes tend to receive the fewest follow-up health care services [29]. In exploring reasons for non-attendance at services, a small number of studies have considered risk perceptions and health behaviours in women with prior GDM. In terms of risk perception, evidence suggests that women with prior GDM tend to have an accurate perception of the general risk of developing type 2 diabetes [30,31]. Yet, despite understanding the association between GDM and future diabetes, women with prior GDM hold inaccurate perceptions of their *personal* risks of the disease. Even those women at the highest risk of developing type 2 diabetes typically do not perceive *themselves* to be at an elevated risk as they intend to modify their health behaviours in the future [30-32].

Notably, studies have shown that having an intention to engage in health behaviour modification does not always translate into actual changes in health behaviours. For example, Kim *et al*. [30] and Doran and Davis [33] found that women with prior GDM who perceived themselves to be at high risk of developing type 2 diabetes *and* planned to modify their future health behaviour were also associated with the lowest fruit and vegetable consumption, and self-reported no physical activity (or any other recent modifications to their health or lifestyle behaviours). A small number of studies have used qualitative methodologies to explore the barriers to and facilitators of post-partum follow-up care in a cohort of women with recent GDM. Bennett *et al.*[34] found that feelings of emotional stress due to adjusting to a new baby and fear of receiving a diabetes diagnosis were the primary barriers to accessing post-partum follow-up care. In fact, women in their study reported preferring a state of unknowing to continuing with the necessary behavioural changes, such as dietary restrictions and blood monitoring, required to reduce diabetes risks. Fear of receiving bad news has been reported as a barrier to participation in other intervention trials too [35].

In an effort to determine the specific factors that could improve attendance and participation in diabetes prevention programmes, Daspupta *et al*. [36] invited women within five years of a GDM diagnosis to a focus group discussion with the aim of delineating factors that could enhance participation and engagement in diabetes prevention programmes. Tellingly, of the 1201 women who received invitations to participate in the study, only 120 contacted study personnel (10%) and only 29 women actually attended one of the four focus groups. Discussants expressed a need for social support to achieve changes in dietary and physical activity habits. In this regard, face-to-face interactions with peers and professionals were preferred, with adjunctive roles for the Internet/social media. Furthermore, direct participation of partners/spouses in type 2 diabetes prevention programmes was viewed as important to enhance support for behavioural change at home. Discussants highlighted work and child-related responsibilities as potential barriers to participation, and emphasised the importance of childcare support to allow attendance.

Despite this instructive body of work, there is little information about the feasibility of offering a lifestyle intervention in a community setting with a population of women with a recent history of GDM. Further research is required to understand the characteristics of women who participate in behaviour modification programmes and whether they differ from non-participators in terms of lifestyle, health status or family history of diabetes. Additionally, we need a better understanding of the specific difficulties faced by women with recent GDM in participating in diabetes prevention trials and programmes. Without such understanding, we have little evidence to draw from to enhance participation of this particular group when planning future RCTs and interventions.

In Ireland, the Atlantic Diabetes in Pregnancy (Atlantic DIP) programme was established in 2005 to investigate screening strategies, interventions and follow-up of women with GDM with a view to diabetes prevention in the long-term [1]. In routine clinical practice in Ireland there is no lifestyle modification intervention for women with prior GDM to reduce the risk of developing type 2 diabetes. In this context, we designed a randomised controlled trial to evaluate the feasibility and effectiveness of an established community-based lifestyle and risk factor modification intervention, the Croí MyAction programme, compared to standard health care for delaying diabetes onset in women with recent GDM [37].

Croí MyAction is a 16 week cardiovascular prevention programme with an emphasis on lifestyle modification (smoking cessation, healthy food choices and physical activity), medical risk factor management (blood pressure, lipids and glucose) and the prescription of cardio-protective medication where appropriate. The programme is nurse-led and delivered by a specially trained multidisciplinary team (MDT) which includes a dietician, physiotherapist and physical activity specialist, supported by a physician. The MyAction model of care evolved from the EuroAction study [38], which demonstrated that a nurse-managed, multidisciplinary, and family-based programme could achieve healthier lifestyle changes and better risk factor control than usual care at one year. Subsequently, Croí MyAction was established as a partnership between Croí (an Irish heart and stroke charity) and Imperial College London, the co-ordinating centre for the EuroAction study [39]. The Croí MyAction programme has demonstrated striking results so far in terms of improving adherence to a healthy diet, reducing both obesity and central obesity and also significantly improving physical activity levels [40] – all interventions which have been shown to reduce risk of progression to type 2 diabetes. While Croí MyAction is designed for individuals at high risk of developing cardiovascular disease we anticipate that it will be similarly effective in reducing long-term diabetes risks in women with prior GDM.

The RCT is currently underway. However, trial recruitment proved far more difficult than initially anticipated despite the promise of a free-of-charge lifestyle intervention for reducing progression from GDM to type 2 diabetes (Croí MyAction). This paper reports on (i) differences between a subset of participants and decliners in our trial; and (ii) the reasons for non-participation cited by all decliners. Through this research we hope to foster a better understanding of barriers to and facilitators of trial participation for women with prior GDM, and in the process support the development of interventions that are more relevant, effective and successful in recruiting the desired population.

## Methods

Our study design, recruitment process and intervention arms are described elsewhere [37]. Briefly, we invited 410 women with prior GDM to participate in a RCT of the Croí MyAction lifestyle intervention programme (full clinical research ethics committee approval was obtained on 27 March 2012 with an amendment approved on 1 September 2012). Of the total 410 women invited to the trial, 89 (or 22%) agreed, 156 declined (38%), and the remainder were not contactable. Among those women who agreed, 35 did not meet our eligibility criteria of glucose dysfunction at baseline and were excluded from the study. Thus a total of 54 women with a history of GDM and persistent post-partum glucose dysfunction [impaired glucose tolerance (IGT) or impaired fasting glucose (IFG)^a^], have been randomly assigned to a control arm (n = 27) or to the Croí MyAction intervention group (n = 27). Women in the control arm receive usual health care advice, defined as written information on diet and lifestyle changes for reducing diabetes risks and visits with general practitioners as required. The intervention group receives usual health care as per the control group in addition to attending the 12-week Croí MyAction lifestyle modification programme. Croí MyAction comprises a group exercise programme, group health promotion or education seminars, and one-to-one meetings with a multidisciplinary health care team focusing on personalised risk factor reductions.

For this paper, we wanted to examine the characteristics of the participants versus the decliners to isolate any key differences that might be predictive of participation. Upon analysis of the available dataset, we identified 43 out of the 89 participants and 73 out of 156 decliners who had returned for follow-up testing 3-5 years post-partum [41]. The statistical analyses in this paper draw from the clinical, anthropometric and demographic characteristics of this sample of 116 women. In addition, we summarise barriers to participation in the trial from the total number of decliners (n = 156). All of these women are part of an ongoing research study on gestational diabetes known as Atlantic Diabetes in Pregnancy (DIP) programme, which has been running since 2005. Each woman has given her consent in writing for her personal health data to be used as part of the ongoing research programme. The women are free to decline to participate in any particular phase of the research. We obtained ethical approval from the Clinical Research Ethics Committee of Galway University Hospital for the conversion study [41] in March 2011, and for the clinical trial in 2012.

We hypothesised that there would be differences between participants and decliners to the trial based on known type 2 diabetes risk factors, and that we would be able to predict probability of participation based on these risk factors. Therefore, the primary response variable of interest is whether a person agreed to participate in the intervention or not. The explanatory variables include the following known type 2 diabetes risk factors: first degree relative with diabetes mellitus (DM); smoking status; exercise participation; daily fruit and vegetable intake; insulin use during pregnancy; post-pregnancy impaired fasting glucose (IFG); post-pregnancy impaired glucose tolerance (IGT); fasting plasma glucose (FPG) value and 2-hour plasma glucose (PG) value, 3-5 years following GDM; body mass index (BMI); and the covariate, age at delivery.

To test our hypotheses, two different approaches were used. First, in order to describe the variables in question and to identify any differences between participants and decliners on those variables, we undertook univariate analysis using Chi-square and T-tests. The Chi-square analysis investigated associations between participants and decliners based on the following categorical variables: family history of type 2 diabetes (yes/no), current smoking status (yes/no), meets daily exercise intake requirements (yes/no), meets daily fruit and vegetable intake requirements (yes/no), insulin use during GDM pregnancy (yes/no), and whether blood glucose values met the diagnostic criteria for either or both of IFG or IGT on a post-partum oral glucose tolerance test (OGTT) (yes/no). T-tests were used to compare the participants and decliners across the continuous variables of: FPG values, 2-hour PG values, BMI and age at delivery.

Secondly, in order to identify variables that may be useful predictors of participation we undertook multivariate analysis, in the form of: logistic regression analysis (using variable selection procedures and the least absolute shrinkage and selection operator [LASSO]; and classification tree analysis (pruned on misclassification). All statistical analyses were carried out with Minitab (version 16) and R (version 2.15) software, using rpart, rpart.plot and beanplot libraries. Model checking was based on deviance and the Hosmer-Lemeshow test. Finally, we summarised the stated barriers to participation available from the 156 decliners.

## Results

### Comparison of participants to decliners

#### Chi-square analysis and T-tests

The Chi-square test of independence was performed to determine if participants and decliners are distributed differently depending on their health status, using Fisher’s exact test when appropriate. The results of this analysis are presented in Table 1. Only one significant association was found: women that used insulin during the index GDM pregnancy were associated with *lower* participation rates than those that did not use insulin in pregnancy: **χ**^**2**^ (1, *N* = 116) = 6.947, *p* < .01. Of the total number of women who used insulin, only 22% agreed to participate in the trial (77% declined); whereas 46% of those who did *not* use insulin agreed to participate (54% declined).

**Table 1:** Chi-square analysis of association

**Categorical variables**				
	**Consented to participate n = 43 (37%)**	**Declined to participate n = 73 (63%)**	**All (n = 116)**	**P-value χ**^**2 **^**test**
First degree relative with diabetes mellitus				
No	22 (35%)	41 (65%)	63	0.601
Yes	21 (40%)	32 (60%)	53	
Smoker				
Past	17 (41%)	25 (59%)	42	0.790
Current	7 (39%)	11 (61%)	18	
Never	19 (34%)	37 (66%)	56	
Meets daily exercise requirements				
No	5 (31%)	11 (69%)	16	0.604
Yes	38 (38%)	62 (62%)	100	
Meets daily fruit and vegetable requirements				
No	0	3(100%)	3	0.178
Yes	43 (38%)	70 (62%)	113	
*Insulin use during pregnancy*				
*No*	*33 (46%)*	*38 (54%)*	*71*	*0.008 95% CI (7.5, 41)*
*Yes*	*10 (22%)*	*35 (77%)*	*45*	
IFG at follow-up				
No	34 (36%)	61 (64%)	95	0.544
Yes	12 (57%)	9 (43%)	21	
IGT at follow-up				
No	41 (38%)	68 (62%)	109	0.631
Yes	2 (29%)	5 (71%)	7	

T-tests of differences between the 43 participants and 73 decliners are presented in Table 2. There was no evidence against normality for the continuous variables. The groups differed significantly on one variable: age at delivery. On average, participants were 2 years older. Specifically, the mean age at delivery for participants was 36 years while the mean age at delivery for decliners was 34 years. There was no evidence against normality for the continuous variables in question.

**Table 2:** T-tests of differences between participants and decliners

**Continuous variables**				
	**Consented to participate (n = 43)**	**Declined to participate (n = 73)**	**95% Confidence interval (CI), Consent-No consent**	**P-value**
	**Mean (SD)**	**Mean (SD)**		
Fasting plasma glucose at follow-up	5.3 (0.63)	5.2 (0.71)	(-0.34, 0.17)	0.526
2-hour plasma glucose at follow-up	5.4 (1.55)	6.3 (2.37)	(-0.06, 0.11)	0.619
BMI at follow-up	33.3 (5.98)	32.1 (6.59)	(-3.52, 1.24)	0.344
*Age at delivery*	*36.1 (4.48)*	*34.1 (5.05)*	*(0.21, 3.8)*	*0.029*

### Predicting probability of participation

#### Binary logistic regression analysis

Logistic regression analysis using variable selection and the LASSO was used to identify useful predictors of participation in the trial. The analysis identified insulin use during pregnancy and age at delivery as the only significant predictors of the probability of participation. The ‘meets daily fruit and vegetable intake’ variable was not included in the logistic regression analysis due to small cell counts.

**Figure 1:**
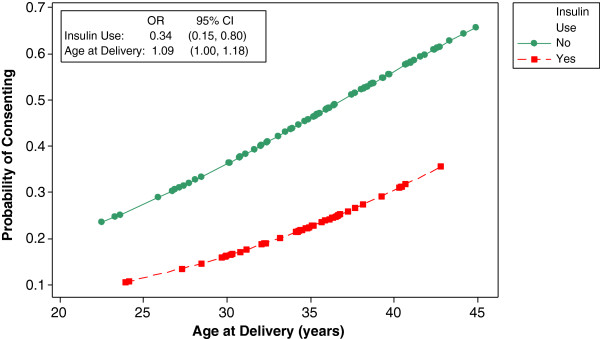
Scatter plot of predicted probability of participation.

Table 3 shows the model estimates from both the full and reduced models. The reduced model identified two useful predictors; namely, age at delivery, and whether an individual was on insulin or not during pregnancy. From the table it can be seen that the estimated odds of participating increase by 1.12 per unit increase in age; and are 0.35 times lower for insulin users compared to non-insulin users. The odds ratio, however, gives no indication of the underlying probability of an individual participating in the trial. Rather, it provides the multiplicative effect on the odds based on a reference category (for a categorical predictor) or per unit increase (for a continuous predictor). Thus, a plot of the corresponding estimated probability is needed to highlight the effect of increasing age for insulin and non-insulin users (Figure 1).

**Table 3:** Full and reduced binary linear regression models of consent to participate

	Full model*				**Reduced model (LASSO)**			
**Variable**	**OR**	**Lower**	**Upper**	**P-value**	**OR**	**Lower**	**Upper**	**P-value**
Age (years)	1.12	1.01	1.23	0.02	1.09	1.00	1.18	0.05
First degree relative								
(Yes vs. No)	0.77	0.32	1.85	0.55				
Smoker								
(Past vs. Never)	1.91	0.72	5.06	0.18				
(Current vs. Never)	1.65	0.46	5.93	0.43				
Exercise								
(Yes vs. No)	0.98	0.27	3.49	0.97				
Insulin use								
(Yes vs. No)	0.35	0.14	0.86	0.02	0.34	0.14	0.82	0.01
IFG at follow-up								
(Yes vs. No)	1.27	0.43	3.77	0.66				
IGT at follow-up								
(Yes vs. No)	0.59	0.09	3.94	0.58				
BMI	1.04	0.97	1.11	0.26				

C statistic: Full = 0.71, Reduced = 0.68.

*Fruit and vegetable intake excluded due to sparse data (113/116 had adequate fruit and vegetable intake).

#### Classification tree analysis

**Figure 2:**
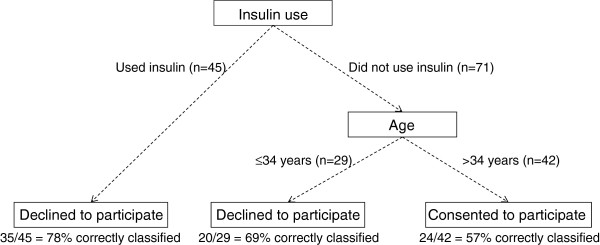
Classification tree for participation.

A classification tree (pruned on misclassification) was grown to identify useful cutpoints for the potential predictors of participation (Figure 2). The (pruned) classification tree identified insulin use and age at delivery as useful predictors; that is, those women using insulin during the index pregnancy, irrespective of age, were classified as decliners; those women not using insulin and > 34 years were classified as participators; and women not using insulin and ≤ 34 years were classified as decliners.

The predictive ability of the tree is assessed by looking at the proportion of women correctly classified by the tree. Figure 2 shows the number of women correctly classified and the corresponding percent correctly classified at each (terminal) node. For example, if insulin use alone was used as a classifier for non-participation, 78% (of those taking insulin) were correctly classified. Those not using insulin during pregnancy and > 34 years were classified as participants, with a correct classification rate of 57%. Those that did not use insulin during pregnancy and ≤ 34 years were classified as decliners, with a correct classification rate of 69%. Overall, 79 of the 116 women (68%) were classified correctly by the tree model.

### Comparison summary

In summary, there were no significant differences between participants and decliners on lifestyle variables (smoking behaviour, healthy eating behaviour and exercise behaviour) or in terms of their diabetes status (IFG, IGT, FPG, 2-hour PG), BMI, or family history of diabetes. Participants and decliners differed significantly on two variables: age at delivery (with women older than 34 years being more likely to participate) and insulin use for glucose control during pregnancy (with women requiring the use of insulin less likely to participate). In terms of predicting participation, the evidence suggests that, for this sample, the factors significantly predictive of probability of participation are age at delivery and insulin use during pregnancy. However, the model gives no indication as to which split in age is most informative. The classification tree suggests that the effect of age is most informative when considering individuals above and below 34 years of age. It should be noted, however, that neither the logistic model nor the tree are intended to be used as prediction algorithms. Rather, they help to identify potentially useful predictors and potentially useful cutpoints for continuous predictors. The cutpoint identified here supports the existing evidence that older women are more likely to participate in research than younger women [31].

### Barriers to participation cited by decliners

Stated barriers to participation in the trial are available from the 156 decliners and are described in Table 4. In total, 251 statements were available for coding as some women cited several reasons for declining to take part in the intervention. Coded statements were grouped into themes that reflected similar or related barriers.

**Table 4:** Barriers to participation cited by decliners

**Barrier category**	**Description**	**Number of times cited (and percentage of respondents who cited barrier)**
Travel distance/Transport	Distance to location of intervention; travel expense; lack of transportation	80 (51%)
Child care commitments	Cannot leave children; do not *want* to leave children; children too young to leave; lack of childcare; leisure time dedicated to children/family activities	52 (33%)
Lack of time/Too busy	Lack of time to commit to the programme; time commitment required too great; unable to commit to *specific* times; own and partners’ work schedules; child’s school and activity schedule	48 (31%)
Research and intervention deterrents	Research fatigue; discomfort with test procedures; programme times/content	26 (17%)
Not concerned about diabetes risks	Given the all clear on post-partum tests, therefore not concerned about risks	23 (15%)
Lack of social support	Lack of a network of friends or family to provide practical or emotional support	9 (6%)
Already taking action on own	Does not need this programme as taking action on her own	8 (3%)
Health too poor to participate	Acute health problems take precedence over chronic health problems	3 (2%)
Other reasons	Planning a pregnancy; caring for parents; learning English; currently living abroad	6 (4%)
No reason given	Not interested in participating	4 (3%)

Above all, travel distance, child care commitments and lack of time to devote to the programme presented the most significant barriers to participation for this cohort of women. This finding is not surprising given the target population: women with recent GDM, many of whom have other children and also work outside the home. Indeed, such barriers have been shown to be particularly relevant to women [34,35]. Many of the women invited to our trial were from rural areas up to a 2-hour drive from the intervention site. For these women, the travel distance and expense, in addition to the length of the programme, was a prohibitive factor. A number of women simply stated that they had children at home and therefore could not attend the programme, some qualifying this statement by saying that they did not *want* to leave the children but would prefer to spend any spare time they had with their children; others said their children were too young to leave. Many specified that, with the demands of their own schedules and their partners’ schedules, they could not commit to being able to leave the family at a regularly scheduled time each week.

Research fatigue also emerged as a significant reason for non-participation for the population of women targeted in this study. This finding may reflect the fact that an intensive programme of research on the issue of GDM has been ongoing in the region for over five years, and the women invited to participate in this trial had all been involved in earlier phases of the research. Moreover, one of the baseline tests - the Oral Glucose Tolerance Test - requires fasting for a minimum of 8 hours prior to the test, then 3 blood draws over the course of 2 hours. This procedure in itself presented a barrier to participation for some women. On the other hand, a significant number of women were not concerned about their health or diabetes risks and felt they were not in need of the intervention programme as they intended to take action to improve their health. This finding affirms those of other studies on women with GDM [30]. Finally, although lack of social support was only explicitly identified by a small number of women as a reason for declining to take part in the trial, it appeared to be an important confounding factor in many of the other barrier categories too. Essentially, if there was no one else to assume the responsibilities of running the home and caring for children, it felt prohibitively difficult for many women to commit to a time-intensive intervention programme requiring a regular commitment of time.

## Discussion/conclusions

Of a fairly large potential candidate population, relatively few women who received information about the trial agreed to participate. For the following reasons we expected uptake to be higher:

•The intervention (Croí MyAction programme) is delivered in an accessible, non-medical community setting, free-of-charge to participants;

•The intervention offers flexible options for delivery (for example, a home-based option with fewer on-site visits is available, if necessary) and allows participants to make up missed sessions on future programme dates;

•The intervention combines individual and group-based components, and encourages participants to enrol a friend or family member as a full participant for social support and motivation outside programme times;

•The program is multidisciplinary, recognising that changing lifestyle and health behaviour requires the expertise of a variety of health care professionals;

•The intervention has very high uptake, retention and success rates with its current target population (individuals at high risk of cardiovascular disease).

Additionally, since the initiation of the Atlantic DIP research programme in 2005, health care providers in our region have increased their focus on providing accessible and relevant clinical recommendations to women with GDM during all antenatal visits. Thus it was expected that women would be motivated to improve their health following GDM.

Overall, the results do not support our hypothesis that participants and decliners differ significantly in their diabetes risk profiles. In fact, in this population, the groups did not differ significantly on lifestyle variables (smoking behaviour, healthy eating behaviour and exercise behaviour), diabetes status (IFG, IGT, FPG, 2-hour PG), BMI, or family history of diabetes. Participants and decliners differed significantly on only two variables: age at delivery (with women older than 34 years being more likely to participate) and insulin use during pregnancy (with women requiring the use of insulin less likely to participate).

Our finding on age is consistent with other studies (for example, see [31]) which suggest that older women tend to participate in prevention studies more frequently than younger women. The finding on insulin use, however, was contrary to our expectations and previous research [30,31]. We hypothesised that women requiring insulin during their GDM pregnancy would be more cognisant of the severity of diabetes and their personal risks of developing type 2 diabetes and therefore more motivated to avoid the disease. However, the opposite was found in this sample. Interestingly, Bennett *et al.*[34] found that women who were at highest risk of developing diabetes, based on antenatal glucose testing and requirement of insulin during pregnancy, appeared to receive the fewest post-partum follow-up care services. Bennett’s study concluded that women’s concerns about their post-partum and future health actually prevented them from accessing follow-up medical care and monitoring. At this point in our study, we are uncertain if similar anxieties associated with receiving a diabetes diagnosis may have been a deterrent to participation. We are conducting interviews with participants in our trial upon completion of the intervention to explore their pre- and post-programme health concerns. If time permits, we also intend to invite a subset of the decliners for telephone interviews to further explore their reasons for non-participation.

The uptake rate for our study was low: of the 410 women invited to participate in the trial, only 89 (22%) agreed. As summarised in Table 4, many of the women invited to participate in the trial simply did not consider the intervention accessible, affordable or practical given their lifestyle and carer responsibilities. To put this in context, the average uptake rate for the Croí MyAction (cardiac care) programme is 93%, and the adherence rate (87%) and 1 year retention rate (94%) are also extremely high [40]. However, these patients are mostly older than our population, and include both males and females. The referral method is also different, with attendees typically being referred to Croí MyAction by their physicians.

Evidence suggests that uptake and adherence rates are generally lower in primary than secondary prevention interventions, likely because individuals attending the former have not experienced a life threatening event. The Diabetes Prevention Program (DPP) and the Finnish Diabetes Prevention Study, for example, reported relatively low levels of participation with community-based diabetes prevention interventions [42][21]. Similar problems with recruitment and retention have been reported in the cardiac rehabilitation literature too [43][44], where uptake rates are an average of 44% and drop-out rates range from 30% to 50%. The barriers to participation reported in the above literature are similar to those identified in our study, and include: service-related factors (for example, limited programme capacity or timings of sessions); patient-related factors (e.g. distance to travel, being older, being female, and having comorbidities) [45,46]; psychological barriers such as depression, anxiety and lack of social support [47]; and beliefs about health self-efficacy [48]. Other studies on participation in research have similarly cited childcare and work conflicts as posing barriers [49,50], such barriers are likely to be particularly relevant to women [35].

Overall, the evidence suggests that uptake of lifestyle modification programmes is challenging for any segment of the population but particularly so for women with children. The method of recruitment into the programme also seems important, with physician-led recruitment likely to be more effective than mail-outs or advertising methods. However, the most effective methods for recruiting potential participants vary according to the gender, age, and race/ethnicity of those individuals and ongoing assessment and revision of the recruitment strategy is therefore valuable [51].

### Lessons learned

Women with recent GDM face multiple barriers to lifestyle change. “For them, illness and its management cannot be separated from the broader circumstances of their lives, and many factors compete for the time and attention that treatment regimens require” [52] (p.361). The study we describe in this paper is a small trial targeting behaviour change aimed at diabetes prevention. Like other behavioural interventions, the recruitment process into the trial was relatively demanding in terms of researcher time and resources and the recruitment rate was lower than anticipated. While the trial is not yet complete, a number of lessons to increase recruitment into similar future interventions are already apparent. We share these below with the objective of assisting other researchers planning RCTs and diabetes prevention programmes which are targeting the same demographic of women with recent GDM.

#### Feasibility studies

Health behaviours are clearly influenced by complex and contextual social, economic and cultural factors, values, beliefs and constraints [53]. As such, to maximise participation in a study aimed at lifestyle modification and behaviour change, the design and implementation of the intervention needs to give serious consideration to the practicalities of the everyday lives of the target participants. Preliminary research (for example, qualitative studies, feasibility studies or pilot trials) should be undertaken to identify these practical details and determine whether the trial is feasible for the target population.

#### Address logistical barriers to participation

Historically, women have been under-represented in clinical trials for various reasons [54-57]. As ours and other studies have shown, the non-participation of women is strongly influenced by logistical issues related to childcare, work responsibilities, travel time and expense, amongst others [34,35,49,50,55]. Given these barriers, to maximise the recruitment of this demographic into RCTs and lifestyle interventions, issues of convenience, relevance and carer-responsibilities must be addressed. This includes, for example, offering home-based study assessments; providing on-site child care facilities; ensuring a wide variety of times and modalities for the intervention; and offering the intervention locally. In general, Hunt *et al.*[52] instruct that “future intervention[s] need to go further in balancing clinical efficacy with the various and competing nonmedical factors that women with GDM confront on a daily basis, such as the demands of employment, family and economic obligations” (p.362).

#### Appropriate trial design

Lifestyle intervention trials differ from traditional treatment trials as participants in the former are *at risk* of a condition rather than living with a diagnosed condition [58]. Lifestyle intervention designed to prevent a disease or condition requires the considerable time, commitment and motivation of participants. Moreover, patients often have strong preferences for one treatment over another. For these reasons, Brewin and Bradley [59] argue that such trials should be conducted differently from treatment trials. Their argument is compelling; in essence, if the effectiveness of a lifestyle or behavioural intervention is evaluated after random administration of patients (who may or may not desire the treatment) it will be difficult to distinguish between a treatment that failed because it was not inherently effective and one that failed because it was not targeted towards patients who were suitably motivated. “Problems of interpretation are bound to arise when trials of participative treatments are designed as though they are drug trials without considering the different psychological processes concerned in each” [59] (p.315). Thus, random allocation – which is considered a great strength in traditional RCTs – can be considered a weakness in lifestyle/behavioural intervention trials because it eliminates participant choice. Choosing one’s preferred intervention, it is argued, can increase motivation and thereby the success of the intervention [53,60]. One solution here may be to adopt a non-randomised approach which capitalises on, rather than ignores, motivational differences between participants by allowing patient choice to dictate the intervention group rather than chance allocation. Such studies have the potential to tell us whether treatment interventions are viable options for patients who choose to use them.

Alternatively, rather than abandoning the RCT completely, realistic compromises should be considered during the design stage in order to increase the potential attractiveness of a trial to participants. For example, patient-preference RCTs – while not without their own limitations – are widely used [61], or simply ensuring that the control arm of an intervention trial is an attractive option for participants while maintaining investigator equipoise about which intervention is most effective.

#### Risk perception and behaviour change

As ours and other studies suggest (see [30,33]), awareness of GDM as a risk factor for type 2 diabetes may not be sufficient to increase personal risk perception and motivate women to change their lifestyle and health behaviours. Other factors such as emotional stress and fear of diagnosis have been shown to present significant barriers to participation in follow-up health care programmes. There is evidence too that women with GDM have higher levels of perceived pregnancy “hassles” and higher rates of clinical depression [7]. The extent to which this persists after childbirth and between pregnancies is not known, but it is likely that worry about subsequent pregnancies is present for many women who had GDM. To address this, health care providers and researchers must ensure they are providing sufficiently reassuring information about how GDM can be successfully managed in order to alleviate concerns among the potential population of participants.

Finally, it seems instructive to note that while individual risk perception does not always result in actual behaviour change, it may still indicate a *readiness* to change [31]. In this respect, researchers should consider integrating cognitive behavioural strategies into lifestyle interventions not only to address the underlying psychological barriers that mitigate success in regard to behaviour change, but also to assist women who are still ‘pre-contemplators’ in regard to lifestyle modification to progress towards the stages of contemplation and taking action [62].

In the end, lifestyle intervention in people at high risk for type 2 diabetes can result in sustained changes and a corresponding reduction in the burden of the disease well after the intervention stops [23]. As such, it is imperative to understand the motivators and barriers to participation in such interventions for women with prior GDM. Our study team has learned a number of practical lessons from recruitment into the current trial. We plan to incorporate these lessons – along with the analysis of trial outcomes – into the design of a larger trial of a revised intervention, anchored in evidence of what works best for this cohort of women in the Irish context.

## Endnotes

^a^The glucose levels used to diagnose IFG and IGT in this study are based on the 2010 recommendations of the American Diabetes Association. GDM was diagnosed based on the recommendations of the International Association of Diabetes and Pregnancy Study Groups (IADPSG) [63].

## Abbreviations

BMI: Body mass index; CI: Confidence interval; DIP: Diabetes in pregnancy; DM: Diabetes mellitus; DPP: Diabetes Prevention Program; FDPS: Finnish Diabetes Prevention Study; FPG: Fasting plasma glucose; GDM: Gestational diabetes mellitus; HRB: Health Research Board; IADPSG: International Association of Diabetes and Pregnancy Study Groups; IFG: Impaired fasting glucose; IGT: Impaired glucose tolerance; LASSO: Least absolute shrinkage and selection operator; MDT: Multidisciplinary team; NGT: Normal glucose tolerance; OGTT: Oral glucose tolerance test; PG: Plasma glucose; RCT: Randomised controlled trial; SD: Standard deviation.

## Competing interests

The authors declare they have no competing interests.

## Authors’ contributions

JJI and AOD are involved in trial management, recruitment, acquisition of baseline data and analysis of outcomes; both JJI and AOD drafted the manuscript. IG, BMcG, LGG, CON and FPD conceived and designed the study; and IG is also involved in trial coordination. JN was also involved in the design of the study and provided advice on the statistical methods. SC was involved in the protocol development and revision of the manuscript. FPD and BMcG revised the manuscript, and all authors read and approved the final manuscript.

## Authors’ information

JJI is a social anthropologist working as a Post-Doctoral Researcher in Medicine. AOD is a psychologist, also working as a Post-Doctoral Researcher in Medicine. IG is Lead Prevention Nurse and Programme Manager for Croí MyAction at Croí, the West of Ireland Cardiac Foundation, in Galway. BMcG is a Senior Lecturer in Clinical Psychology, Director of the Doctor of Psychological Science programme in Clinical Psychology at NUI Galway, and Joint Director of the Centre for Pain Research. JN is a Senior Lecturer in Biostatistics in the HRB Clinical Research Facility at NUI Galway. LGG is a GP in practice in County Clare, Ireland, and is also founding Clinical Director of the Western General Practice Research and Education Network in Ireland and Senior Lecturer in General Practice at NUI Galway. CON is Professor of Economics at the J.E. Cairnes School of Business & Economics at NUI Galway. SBC is a Consultant Cardiologist in Imperial College London and a member of the original EuroAction steering group. FPD is a Consultant Endocrinologist at the University Hospital Galway; Head of the School of Medicine at NUI Galway; Director of the Galway Diabetes Research Centre and lead Principal Investigator responsible for the scientific and technical direction of the Atlantic DIP research programme.

## Pre-publication history

The pre-publication history for this paper can be accessed here:

http://www.biomedcentral.com/1471-2288/14/13/prepub
